# The Healthy Pregnancy Service to Optimise Excess Gestational Weight Gain for Women with Obesity: A Qualitative Study of Health Professionals’ Perspectives

**DOI:** 10.3390/jcm9124073

**Published:** 2020-12-17

**Authors:** Rebecca F. Goldstein, Ruth E. Walker, Helena J. Teede, Cheryce L. Harrison, Jacqueline A. Boyle

**Affiliations:** 1 Monash Centre for Health Research and Implementation, School of Public Health and Preventive Medicine, Monash University, Clayton 3168, Australia; rebecca.goldstein@monash.edu (R.F.G.); ruth.walker@monash.edu (R.E.W.); helena.teede@monash.edu (H.J.T.); cheryce.harrison@monash.edu (C.L.H.); 2 Diabetes and Vascular Medicine Unit, Monash Health, Clayton 3168, Australia; 3 Monash Women’s, Monash Health, Clayton 3168, Australia

**Keywords:** gestational weight gain, obesity, midwives, obstetricians, health coach, intervention, implementation, qualitative, health professionals

## Abstract

Maternal obesity is associated with health risks for women and their babies, exacerbated by excess gestational weight gain. We describe health professionals’ perspectives in the provision of a Healthy Pregnancy service designed to optimise healthy lifestyle and support recommended gestational weight gain for women with obesity. Semi-structured interviews were conducted with health professionals. Questions were based on the Theoretical Domains Framework (TDF) and deductive thematic analysis was performed. A total of 14 multidisciplinary staff were interviewed. Six themes were identified: 1. health professionals view themselves as part of a team; 2. health professionals reported having necessary skills; 3. experience generated confidence in discussing gestational weight gain; 4. gestational weight gain is considered of variable importance; 5. health professionals want women to be comfortable; 6. the environmental context and resources presented some barriers. Staff were supportive of the Healthy Pregnancy service and valued developing teamwork with staff and rapport with women. Most felt relatively comfortable discussing weight gain with women. Barriers included ability to navigate sensitive topics with women, limited awareness of the intervention among new staff, communication between teams, and waiting time for women. Barriers and enablers to the delivery of an integrated model of maternity care were identified. These findings should inform and improve implementation of service models integrating healthy lifestyle in the antenatal care of women with obesity.

## 1. Introduction

High pre-pregnancy body mass index (BMI) and excessive gestational weight gain (GWG) both independently contribute to adverse maternal and neonatal outcomes [1,2], as well as to increased risk of postpartum obesity in mothers and their children [3,4]. The National Academy of Medicine (previously Institute of Medicine, IOM) recommendations for healthy GWG are specific to a woman’s pre-pregnancy BMI [5]. A systematic review and meta-analysis [6] of more than one million women revealed that almost half gained above GWG recommendations, leading to adverse maternal and neonatal outcomes. Women above a healthy weight preconception had the highest prevalence of excess GWG [7]. Lifestyle interventions prevent excess GWG and improve maternal and neonatal outcomes [8].

Australia’s Clinical Practice Guidelines for Pregnancy Care recommend that at every antenatal appointment, women are given the opportunity to be weighed, discuss weight change, diet and level of physical activity [9]. In Australia, most women with obesity have antenatal care in a hospital setting. In antenatal care, regular weighing and information alone do not reduce excess GWG [10], and women need dedicated lifestyle intervention to support healthy lifestyle and improve GWG and health outcomes [11]. Pregnancy is a ‘teachable moment’, when women are more likely to be receptive to positive health behaviours [12,13]. Although studies show women are amenable to making changes in pregnancy, progress can be limited due to health professional barriers including time limitations, lack of training and reluctance to talk about the sensitive issue of gestational weight gain with women [14,15,16], as well as individual barriers of body image, family, work and knowledge [17,18].

In this context, The Healthy Pregnancy service was established in 2015 at Monash Health, the largest health service in Australia, to care for women with a pre-pregnancy BMI of ≥35 kg/m^2^. This co-designed antenatal service is an embedded, evidence-based lifestyle intervention. It is based on the principles of the Healthy Lifestyle in Pregnancy Intervention (HeLPher) [19,20], shown to be effective in women at increased risk of gestational diabetes in routine antenatal care. The program is underpinned by goal setting, self-monitoring, social support and problem solving. The use of dedicated staff including physician (endocrinologist) and health coach to deliver the program addressed the barriers of time constraints and expertise of routine maternity health professionals. The program also created an environment where all health professionals in the service were facilitated to undertake conversations with women around healthy pregnancy with lifestyle advice. This lifestyle intervention program constitutes pragmatic implementation research, where evidence is generated in the context of usual clinical care [21,22,23].

Implementation research has identified 14 domains within the Theoretical Domains Framework (TDF) that are related to behaviour change, allowing for targeting of intervention to increase guideline implementation [24,25]. These domains have been used to understand midwives’ [14,26] and general practitioners’ [15] experience of GWG in pregnant women, but there are limited reports about the TDF domains in multidisciplinary service settings [27].

The aim of this study was to explore the experiences and perspectives of the health professionals working in the integrated antenatal clinic and Healthy Pregnancy service in order to understand the barriers and enablers to the provision of lifestyle change advice.

## 2. Experimental Section

### 2.1. Materials and Methods

#### 2.1.1. Study Design

A qualitative approach assessed health professionals’ experiences while working in the Healthy Pregnancy service. Data was collected via semi-structured interviews with questions based on the Theoretical Domains Framework (TDF) that explores barriers and enablers of behaviour change and implementation research methods [25] (Table 1). The consolidated criteria for reporting qualitative research (CROEQ) 32-item checklist was used in planning and reporting [28]. This study was approved by the Monash Health Ethics Committee.

#### 2.1.2. Clinic Setting

This study is part of a broader pragmatic clinic trial (the Healthy Lifestyle in Pregnancy Project, HiPP) that evaluated the effect of a lifestyle intervention on gestational weight gain and maternal and infant outcomes in women with maternal obesity (Australian New Zealand Clinical Trials Registry: 12620000985987). The project was implemented within a large hospital network in metropolitan Melbourne, Australia, with approximately 10,000 live births per year [29]. Australia offers universal freely accessible health care and Monash Health is the largest health service nationally, situated in a low socio-economic status (SES), diverse ethnic background catchment [30]. The specific service provided care to women with a BMI of 35–43 kg/m^2^ with approximately 200 live births per year, in a collaborative care model with obstetricians and midwives.

The Healthy Pregnancy service embedded a patient-led behaviour change lifestyle intervention, based on principles of the effective Healthy Lifestyle in Pregnancy intervention (HeLPher) [31]. It is delivered by a health coach and a physician/endocrinologist (intervention staff) at five sessions integrated with routine pregnancy care. The intervention is underpinned by social cognitive theory and focuses on behaviour change and self-management of weight, healthy diet and exercise, with skills practiced in goal setting, problem solving and relapse prevention. The first intervention assessment coincided with the first medical review, typically scheduled between 12 and 18 weeks. Session 2 occurred between 20 and 22 weeks, session 3–28 weeks, session 4–32 weeks and session 5–36 weeks. The study design was cognisant of the clinical demands of midwives and obstetricians who are generally time-poor and lack the training to spend prolonged periods counselling women on healthy lifestyle [32]. Therefore, midwives and obstetricians (non-intervention staff) in the Healthy Pregnancy service did not deliver the behaviour change intervention, but were supportive of the program messages and reinforced this to women throughout. Midwives and obstetricians had the opportunity to liaise with the intervention staff about individual’s progress.

In this particular health service in Australia, women with a BMI ≥ 35 kg/m^2^ are required to have their pregnancy care in a hospital setting. Currently, there is no standard national clinical approach for weight management in pregnancy. In this service, our clinical model of care involved a physician in addition to maternity staff. This acted to streamline care within the service, with the physician having a dual role in delivering the intervention and treating any medical conditions that required specialist care (gestational diabetes, thyroid dysfunction).

Administration clerks (non-intervention staff) responsible for all maternity bookings identified eligible participants and booked them either into the Healthy Pregnancy service, or if the weekly booking exceeded capacity or the day of the service was inconvenient, women were booked to alternative services, which comprised the control group. During their first midwife appointment, the midwife briefly introduced the clinic concept and the additional embedded intervention sessions which were integrated into the standard appointment template. Women could also be referred by midwives from other clinics. The comparison group comprised women who received standard care with no embedded lifestyle intervention.

#### 2.1.3. Participants and Recruitment

Participants were recruited by RG (a female clinician-researcher) in person or by email. Purposive sampling targeted multidisciplinary staff working in the service: clinic midwives, nurse unit managers, an obstetrician, physicians, health coach and an administration clerk. Only 2 staff members (midwives) declined the interview due to time restrictions. One of the obstetricians (JB) and the main health coach (CH) working in the service were not interviewed as they were researchers involved in this study. Many of the clinicians also worked in other maternity clinics within the service, caring for women with normal weight to obesity. Participants were asked to answer questions about their experience in the Healthy Pregnancy service only. Staff are collectively referred to as health professionals, any responses from the administration clerk will be explicitly labelled.

Semi-structured interviews were conducted over the phone or in person by two female clinician-researchers (RG and RW), both with postgraduate expertise in qualitative methods. RG worked in the clinic as an endocrinologist for 1 year prior to commencement of the research project and then ceased clinical work to focus on the research. RG chose not to interview the staff with whom she had an existing professional relationship. RW was not employed in the clinic and joined the project in a research capacity only. RG performed the midwife interviews and RW the remainder. Written informed consent was obtained from all participants prior to the interview. To ensure rigour in the interviewing process, the interviewers discussed progression of the interviews at several times throughout the process. The interviews were between 10 and 30 min in duration and conducted in October and November 2019. Data from the interviews was audiotaped and transcribed verbatim by an independent transcribing service. Participant details were deidentified for anonymity. The facilitators modified the line of questioning according to answers for each domain. Interviews were collected until data saturation was reached, determined when no new ideas emerged from the interviews.

#### 2.1.4. Data Analysis

All transcripts were independently analysed and coded by two researchers (RG and RW), using the NVivo 12 software (Nvivo (Version 12), QSR International Pty Ltd., Melbourne, Australia (2018)). Data was searched for concepts in relation to research questions. These were given code names, and code names were categorised according to the TDF in a deductive process of thematic analysis [25]. Codes related to one or more domains. Coding of three randomly selected transcripts were undertaken by the researchers (RG, RW). After cross-checking for consistency, all transcripts were coded before the authors met to finalise coding and agree on theme development.

## 3. Results

Women who attended the Healthy Pregnancy service between 2016 and 2018 were compared to standard care regarding the primary outcome of GWG and secondary maternal and infant outcomes [33]. Intervention uptake was 95%, and 87% of women attended 80% or more of the five sessions. Women’s perspectives of the clinic were also gathered via qualitative interviews and questionnaires (data not presented here).

Overall, 14 female staff members participated in our study (demographics in Table 2): 7 midwives, 2 nurse unit managers, 1 obstetrician, 1 health coach, 2 physicians and 1 administration clerk.

Health professionals’ experiences of working in the Healthy Pregnancy service were related to six domains of the TDF: (1) social/professional role and identity, (2) skills, (3) beliefs about confidence, (4) emotions, (5) beliefs about consequences, and (6) environmental context and resources (Table 3). These themes and how they relate to the TDF are described with example quotes below.

### 3.1. Theme 1. Health Professionals View Themselves as Part of Team to Support Healthy Lifestyle GWG

Health professionals perceive themselves as being part of a multidisciplinary team to promote healthy lifestyle behaviours, and recognise the importance of team work and reflection on feedback and achievements.

- Sub-theme: Staff roles

Midwives identified their roles as referring women to the Healthy Pregnancy service (from within the usual clinic channels or from other clinics), providing initial advice about healthy weight goals, regularly weighing women and supporting lifestyle goals developed with the health coach and physician, recognising that they do not have the time or expertise to deliver more detailed advice.

“as a midwife I would, as I say, initially just refer them, and then throughout the midwife appointments I would just make sure that they are trying to stick to the diet plan that the… health coaches, have put in place” (Midwife #6, junior, limited experience in clinic)

“weighing them throughout pregnancy we can, um, you know, just keep an eye on what their weight gain is and reinforce that advice depending on how they’re going with their weight.” (Nurse unit manager 1, senior midwife)

Nurse unit managers recognise their leadership role as helping the midwives identify women appropriate for the clinic.

“we’ve done lots of education with our teams and our midwives that work in clinic that any patient that comes through that fits that criteria (for Healthy Pregnancy service) needs to be directed into that space.” (Nurse unit manager 2, senior midwife)

- Sub-theme: Teamwork/Communication between staff

During the clinic, there is open communication between the health coach, physician, nurse unit manager and obstetrician as needed, regarding new referrals as they arise or complex women. Issues could arise at times as the obstetricians/midwives and the health coach/physicians use different electronic database systems for recording clinic notes and are not always able to access both. Junior midwives are more likely to address concerns with their nurse unit manager.

I think that from an in-charge perspective we have really good relationships with the… physician and the health coach.” (Nurse unit manager 2, senior midwife)

“I think the health coach and the physician work very closely together. So in terms of the patient flow we were always sort of touching base during the clinic and just seeing where each other are at, who’s seeing which patient. We even touch base about how the patients are going, if there’s anything of note, so yeah. I feel very well supported.” (Physician 2)

“there is some sort of miscommunication using two different (electronic medical record) systems., so I’m still dependent on the obstetrician printing the notes and leaving that in the patient’s obstetric folder, so that I can see what they’ve said, otherwise there’s no way of knowing what they’re doing.” (Physician 1)

- Sub-theme: Staff feedback

Overall, the staff reported positive feelings about the clinic, and acknowledged teamwork and expertise as being the main enablers allowing the obstetricians and midwives to devote their time to other issues. Having the physicians on-site streamlined referrals. They were able to identify some success stories amongst the women that engaged in the service.

“I think initially they (other staff) weren’t sure about our role in the clinic but I think now they’re absolutely delighted to not have to talk about all this stuff themselves. So I think they’re actually very happy to have us there.” (Physician 2)

Staff perceived that their interventions were making an impact on the women’s pregnancy, with women taking on some of the goals discussed.

“And a lot of women say “I wish I had this in my previous pregnancy”. So most people are actually really open to it and, you know, I think you know get a lot of it.” (Physician 2)

“I think they, that most of the people actually took the advice on board and – especially when …we actually brought the gestational weight gain on a chart so that’s…really good feedback for them as well so that they can actually see that their gestational weight gain is sort of off the chart, it becomes a really good.” (Health coach)

“I always think about one girl who...actually only gained six kilos in her whole pregnancy. Um, it wasn’t stressful, there was no like pressure, it was just - just simple, just good education,” (Midwife #3, senior, a lot of experience in clinic)

### 3.2. Theme 2. Health Professionals Reported Having Skills to Run a Healthy Pregnancy Service

Health professionals in the Healthy Pregnancy service (both intervention and non-intervention staff) believed they had the skills to meet the objectives of the clinic and provide the level of care required to meet women’s needs. Key skill strengths included clearly describing the purpose of the clinic to women, developing rapport and supporting them to be involved in their goal setting.

- Sub-theme: Open communication in describing the clinic to women

Midwives felt that if they clearly explained the purpose of the Healthy Pregnancy service to women, then women would be more likely to approach it positively. In addition, most midwives explained that they weighed all women at every appointment, thereby normalising the process. Overall, midwives felt that they had good communication skills in explaining the service to women in an open, clear manner. The establishment of the service with specific goals has enabled conversation about weight gain.

“They tend to be then more open when you tell them why you’re doing it, that it’s not just to be mean.” (Midwife #5, senior, a lot of experience in clinic)

“I think the fact that we ask to do a weight at every appointment can make it easier, because you’re not just singling somebody out to do it. You’re doing it for everyone.” (Midwife #1, senior, a lot of experience in clinic)

“it’s really sort of legitimised in a lot of ways... I don’t want to say the stigma, but it’s made it easier to talk to these women about weight gain because they’re going to—they’re going—they’re in that clinic for that purpose. So it does sort of make it a gentler introduction for me.” (Obstetrician)

- Sub-theme: Develop rapport

Health professionals recognised that their skills in making women feel comfortable created an environment where women were more likely to talk about sensitive issues such as diet, weight and lifestyle and this was facilitated by ongoing relationships through the pregnancy. One midwife drew on her personal weight issues to relate to women.

“we have a nice team involved. I think women are used to us just discussing it in a very, you know, sensitive non-judgemental way. So I think that helps.” (Obstetrician)

“if I get to know them I start building that rapport…they’re happier to talk about it and they’re happy to tell me about their diet and seek help” (Midwife #6, junior, limited experience in clinic)

- Sub-theme: Empowering women to develop healthy lifestyle behaviours

Intervention staff were able to use their coaching skills to help women set and achieve their behaviour-change goals, giving them an active role in decision making, rather than providing prescriptive advice.

“focusing on…getting the people to come up with their own areas of improvement and setting their own goals. So it’s more a patient-focused kind of approach.” (Health coach)

“To actually hold back and, and try and tease that out of the person instead and ask them …what they know about that area and what they think they should be doing. And more often than not, they actually do have a pretty good idea” (Health coach)

“we…encourage the women to work out themselves, to work out new strategies to overcome those barriers and maybe new plans on how to achieve the goal” (Physician 1)

Physician

### 3.3. Theme 3. Experience Generated Confidence in Discussing GWG

Confidence levels around discussing GWG varied significantly amongst the staff, with senior staff in the intervention team feeling confident to deliver their component of the program. Non-intervention staff would still discuss GWG with women, but in a supportive role, rather than in a delivery role. Junior midwifery staff, who may have less experience in discussing weight and weight gain, were less confident.

Physician “I feel confident…I think because I’ve been working in that clinic for the last… three or four years. Like, I’ve worked in it since it started. So, um, I think just having to have those conversations with women.” (Obstetrician)

“a lot of our more junior staff would feel less…(confident).” (Midwife #2, senior, limited experience in clinic)

“I wouldn’t say I’m confident, but I do it because I know that women do need to know about it, yeah.” (Midwife #6, junior, limited experience in clinic)

### 3.4. Theme 4. Health Professionals Want Women to Be Comfortable in This Service

Health professionals are aware of the sensitive nature of talking about obesity and weight gain. They are mindful of women’s feelings and want to provide patient-centred care, offering care women need in a sensitive and non-confronting manner. At the same time, they recognise that some women with high BMI are reluctant to discuss their weight and at times will hold back in order to avoid conflict.

- Sub-theme: Women’s reluctance to engage

Health professionals had realistic expectations about some women’s reluctance to engage with the intervention sessions. The health coach and physician reported that some women are reluctant to engage if they are uncomfortable discussing their weight, or if they feel they have heard the advice in previous pregnancies and can manage on their own. In these cases, caregivers pull back and accept that engagement is optional.

Physician

“There is a small proportion of women who actually felt um, felt almost like she’s been punished by being sent to this service … So none of them actually self-elect to be there and were simply um referred to this particular service because of their BMI…and there is that … almost animosity kind of attitude where, yeah. And I remember one woman in particular, she basically was just really unresponsive” (Health coach)

“I do get some women who just for whatever reason have decided that that’s not for them, um, and so I just address that with them and just make sure that they know what the clinic involves and then move on.” (Obstetrician)

“if they’re really adamant (that they don’t want to engage), like this is what I am and I don’t care basically well then there is no point making a big issue out of it…because you just alienate the women and, um, it makes it harder to engage with them on other topics that are equally important, I guess.” (Nurse unit manager 1, senior midwife)

- Sub-theme: Midwives’ fear of upsetting women

The caregivers are fearful of causing distress to women, and need to balance being mindful of their feelings and delivering the health care they require. In these circumstances, the midwives will pull back.

“Pregnancy’s meant to be a nice, happy time, and I don’t want them to think that I’m judging or - or you know, making judgments on their lifestyle”. (Midwife #2, senior, limited experience in clinic)

“It’s also one of the most challenging things to talk about because, you know, people are sensitive about their weight and you, like you don’t want to come across as though you are judging them, but it’s being able to present it in terms of, um, being a desirable outcome without, like women feeling pressured about it, I guess”. (Nurse unit manager 1, senior midwife)

- Sub-theme: Managing women’s negative experience

A few women described negative experiences with attending the clinic. Senior staff took these criticisms on board, and using feedback and self-monitoring adjusted their behaviour.

“from time to time women will come up and say “I don’t want to make that appointment, I don’t want to see them again”, um, you know sometimes I’ve had a couple of women say “I’m fat, I know I’m fat, I’ve always been fat, nothing’s going to change that, so I don’t want to make a big deal of it”, so “Okay, that’s fine you don’t have to see them if you don’t want to”. (Nurse unit manager 1, senior midwife)

“Initially I, um, when I started the clinic I remember that there were a couple of women who were not very happy with…their first appointment with me… I asked them about…pre-existing weight, history of weight gain, these sort of things and, um, I kind of had a meeting with the health coach and we tried to rephrase, um, you know, those kind of critical questions and addressing their sensitivities, um, and that was it. Since then it’s been all good. (Physician 1)

### 3.5. Theme 5. GWG Is Variably Considered of Importance among Health Professionals

Most of the health professionals viewed excess GWG as a priority in their clinical care. There was variation in the importance placed on maintaining appropriate GWG within the non-intervention senior staff, who at times may need to prioritise other medical problems encountered in pregnancy.

“I know that that can impact more on their pregnancy and their risk of complications. Um, so it is a pretty high priority.” (Midwife #1, senior, a lot of experience in clinic)

“there’s schools of thought on whether or not, you know, weighing women in pregnancy is of any benefit at all…and I know in the past we never used to do it… certainly knowing what the BMI is at the start of pregnancy is of use…I suppose it’s a priority for me because Monash Health stipulates how we practice.” (Nurse unit manager 2, senior midwife)

“I generally tend to look at what—what their weight gain is and I will discuss it if it’s excessive or if it’s minimal but—no. It—it’s not my be all and end all. I think foetal growth is far more important to me” (Obstetrician)

### 3.6. Theme 6. The Environmental Context and Resources Presented Some Barriers

The environmental context and resources were reported by health professionals as barriers to the Healthy Pregnancy service. For many women, the timing and wait time were inconvenient. Midwives also felt the pressure of inadequate time during consultations. As the service continued, midwife awareness of the service and subsequent referrals also declined.

- Sub-theme: Waiting time and clinic day unsuitable for women

One of the biggest barriers to engaging women in the service was the waiting time. At the initial appointment, women were required to see three clinicians: obstetrician/midwife, health coach and physician for a complete medical assessment and treatment plan (thereafter, intervention appointments were with usually either the health coach or the physician, at the clinicians’ discretion). The computer booking system does not enable health professionals to know if a woman is in the waiting room or currently being seen by another clinician. As a result, occasionally women wait for longer than anticipated. This can lead to them leaving in frustration or because they have competing interests (e.g., a frequent issue was the need to pick up children from school). Nurse unit managers suggested that running a second clinic on a different day and in the morning may give greater flexibility and increase the number of women engaging in the service.

“It’s quite difficult to juggle because they’re waiting around for the appointments, seeing a midwife and then the endo aren’t sure if they’ve been seen—we haven’t really figured out an easier way to facilitate that which is probably the biggest challenge that they’re sometimes left waiting for quite a long time.” (Midwife #7, senior, limited experience in clinic)

“it’s quite common that women will be part of the healthy lifestyle program and they’ll come and they’ll have their first appointment and things are running a bit late and they sit there and they haven’t been seen yet and they’ll come to the desk and say “Look I can’t wait any longer I have to go”, um, so that’s yeah, I would say that’s a significant barrier to women accessing the clinic” (Nurse unit manager 1, senior midwife)

Additionally, co-ordinating the bookings can be a challenge for administration. The administration clerk suggested changing the clinic codes may make bookings more streamlined.

“it just is a matter of juggling, um, screens and going into different codes, going back and forth because I always make sure that the appointments are as close together as possible, understandably the women don’t want to like come and see the midwife at 1.30pm and then not be able to get into the healthy lifestyle appointment till 3.30pm… but we can usually make it work.” (Administration clerk).

- Sub-theme: Lack of awareness of Healthy Pregnancy service and content of clinic amongst some midwives

A number of midwives reported many new staff coming through the clinics, who may not be aware of the Service and therefore may not be referring women who are appropriate.

“we’ve lost a lot of our more senior midwives, and not as many midwives are doing clinic. So we’ve got a lot more junior staff coming through...and we’ve got a lot of staff just being trained up and put in, so they’re probably not aware of it.” (Midwife #2, senior, limited experience in clinic)

More widespread awareness about the clinic to midwives working in other clinics was suggested to increase uptake. Midwives and the administration clerk expressed limited knowledge about the content delivered in the intervention, and showed interest in learning more so they can inform women.

“I think probably every—start of every session people need to be reminded, BMI’s over 35, off to healthy lifestyles”. (Midwife #3, senior, a lot of experience in clinic)

“maybe some more transparency to the rest of the staff about what they talk about and… running some education services or information in the tearoom so that, you know, for those women that aren’t in the service the same kind of discussions can be held by other clinicians.” (Obstetrician)

“I’m just wondering if perhaps like the midwives could have some type of in-service as to being more educated on what the service offers… I wonder if it would be good to, yes sort of refresh them as to, um, yeah the service that’s available.” (Administration clerk)

- Sub-theme: Improving patient resources

Staff suggested providing more written resources for women about healthy weight gain, and putting educational information on the TV in the waiting room.

“If we could get some written information for them as well but maybe something more comprehensive and, ah, most women use like different pregnancy applications, but you know, something more generic that we can, um, advise and like suggest to them and to download and use to track their weight gain. Um, something that could make it more visual and objective for them as well.” (Physician 1)

“Instead of just having midday TV running, maybe we could actually utilise that for education purposes. They might listen.” (Midwife #5, senior, a lot of experience in clinic)

## 4. Discussion

This qualitative study evaluating health professionals experiences of the Healthy Lifestyle in Pregnancy Project (HiPP) identified six TDF domains that act as barriers or enablers for the implementation of this service—feeling part of team to support healthy lifestyle and gestational weight gain; having the required skills; experience generated confidence; variable importance of GWG to health professionals; wanting women to be comfortable in this service; and the environmental context and resources. Translation of these findings into practice is important to improve the implementation model and advance knowledge, clinician skills, confidence and resources.

There are a number of qualitative studies in the literature describing the experience of managing GWG in pregnancy for women across the BMI spectrum. However, the majority of these studies either survey midwives alone and/or describe the practice in routine antenatal clinics only [14,23,26,34]. The design of this study, however, is unique in that we describe an intervention embedded within routine care to limit GWG with dedicated intervention staff, and interview the full spectrum of health professionals. There are two studies that describe intervention models in this space: In Davis’ Australian study [35], they offered a group-based intervention for women with a BMI > 25 kg/m^2^ in antenatal care. However, this was facilitated by midwives, bringing in additional staff (dietician, physiotherapist) for specific sessions; only midwives are interviewed. In Jewell’s UK study [17], women with a BMI > 30 kg/m^2^ were offered weekly group-based sessions throughout pregnancy and up to 6 weeks postpartum with a midwife and a commercial weight management consultant; only pregnant women are interviewed. Our model was based on the recognition that those skilled in lifestyle intervention, including dieticians and exercise physiologists, are best placed to deliver these interventions. Hence, this service was delivered by a health coach and embedded alongside routine maternity care, and maternity health professionals reinforced the lifestyle intervention messages. In this way, women had the expertise they needed, without the need for additional appointments, whereas other studies did not have this service ‘in-house’ and its absence was noted [22,32]. To our knowledge, this is the only qualitative study describing such an intervention in a one-on-one setting in a multidisciplinary service.

Health professionals perceived themselves as being part of a multidisciplinary team and recognised the importance of team work and reflection on feedback and achievements. Understanding of staff roles and teamwork are factors that contribute to a cohesive workplace. Here there was clear awareness of staff roles, in contrast to other studies [27] where it was unclear who was responsible for managing weight in the context of shared maternity care, which can create confusion. Midwives and obstetricians are often not trained or supported with adequate time and resources for delivery [36]. This was relevant to the current clinic and hence, midwives and the obstetrician were appreciative of the lifestyle implementation team’s role to deliver an integrated intervention service and the maternity team were able to support women to maintain the plans developed by the health coach and physician.

Overall, staff displayed skills needed to run a Healthy Pregnancy service; they had open communication in describing the clinic to women, developed rapport in a non-judgemental way and encouraged them to be involved in decision making. The nature of the intervention being specifically designed for women with obesity meant that from the outset, the aims of the clinic were clear. As such, women were weighed regularly, in contrast to other studies [22,32,37], and staff were forthcoming with the measurements, unlike variable practice in a US study of midwives and obstetricians [38]. In this fashion, conversation around excess weight gain was already more acceptable among the staff, in contrast to a recent UK study, where conversation around weight was not yet normalised [27].

By interviewing a variety of health professionals, we were able to capture a broad perspective of the service. Confidence in discussing GWG and healthy lifestyle was high among the health coach and physicians given that they were delivering the intervention and developed sufficient experience during the time working in the service. Confidence among the midwives varied, with senior midwives expressing more confidence, in line with another Australian study describing confidence growing over time [35]. Lack of confidence voiced by junior midwives here is in keeping with other studies, due to inexperience communicating about excess weight [14,26,27]. Importance of GWG was viewed differently by health professionals; with the some of the senior staff (obstetrician, nurse unit managers) placing less of a priority on GWG than other staff members as they need to juggle other medical complications that arise.

Health professionals wanted women to be comfortable in this service. Staff members, midwives in particular were at times fearful of upsetting women when discussing the sensitive topic of GWG and obesity. This sentiment is echoed almost universally among health professionals [14,22,27,32,39,40,41], including the Australian intervention study of staff responsible for recruiting women into a similar new service model [35]. A probable contribution to this fear may be related to minimal undergraduate teaching in medicine and nursing, of skills related to weight gain conversations in pregnancy [15,42]. This view was not expressed by the intervention staff because they have more experience in dealing with this due to the nature of their roles; they are skilled with how to broach these conversations in a sensitive way that still enables rapport to be built. This has identified an area for further upskilling for all staff, in matters of dealing with sensitive issues (for fear of affecting relationships) and negative feedback.

We also identified some barriers in the environmental context that could be improved. Some newer booking staff members had less knowledge of the clinic and were referring less often; more regular education about the clinic would be of benefit. Non-intervention staff did express interest in learning more about what is implemented, which may in turn strengthen their support of the women, highlighting the need to keep them informed especially as new staff change over. In some situations, when staff members did not have access to the correct databases, communication between providers was not ideal. Staff reported a longer waiting time and clinic day being unsuitable for women as limiting factors in service uptake; this has been previously described [35] and needs to be addressed in future clinic design, with more options offered to women. Additionally, health professionals suggested improving patient resources, either in the form of electronic applications or information provided in the waiting room. This needs to be considered in the context of women’s preferences, which will be analysed in a further stage of this project. However, it should be noted that feedback of these study findings to health professionals and women, and contributing to a co-design approach of future implementation may help overcome these barriers [43].

### Strengths and Limitations

To the best of our knowledge, this is the only study that has evaluated a patient-led pragmatic intervention, delivered in the maternity setting and gaining perspective from staff members with different staff roles, allowing a more balanced perspective of the intervention. The findings gleaned have a key role in informing guideline implementation and scale up of interventions in this space. The study design was strengthened by the theoretical domain framework designed to explore guideline implementation [25]. The findings of this research will be strengthened when paired with patient perspective. The results are limited by the participation of only one of the health coaches and obstetricians; the other clinicians were not interviewed as they were researchers in this study. RG’s clinical experience may have influenced her interpretation of the participant’s response. However, reflexivity was considered in the research process to increase rigour [44]. Additionally, this experience is of a single clinical service in one health service and will need to be interpreted with this in mind.

## 5. Conclusions

As excess GWG and obesity in pregnancy are prevalent and lifestyle intervention in antenatal care is effective, implementation knowledge is vital to inform and evaluate appropriate services aimed at improving outcomes. In this HiPP study, we have identified some barriers and enablers to the delivery and uptake of a lifestyle intervention, as perceived by health professionals. Translation of these findings into practice is important to improve the implementation model and advance knowledge, clinician skills, confidence and resources.

## Figures and Tables

**Table 1:** Semi-structured interviews questions mapped to TDF.

Domain	Question
Knowledge (All interviewees)	What do you know about the Healthy Pregnancy service? What is the purpose of the Healthy Pregnancy service? What is your understanding of how women are referred to the Healthy Pregnancy service?
Skills (Health professional interviewees—not the administration clerk)	When are you likely to talk about GWG with women? Do you initiate conversations about GWG with women or do they? If you notice increased GWG, do you ask women about it? If so, how do you go about this? Do you feel you are delivering GWG information consistently? Why/why not?
Social/professional role and identity (Health professional interviewees—not the administration clerk)	What do you see as your role in the Healthy Pregnancy service? Are discussions about GWG and healthy lifestyles within your professional role? Do you do anything different in this clinic compared to the other clinics you work at? Do you weight women or talk about weight with women them?
Beliefs about capabilities (Health professional interviewees—not the administration clerk)	Do you feel confident talking about GWG with women? Do you believe you have the skills to talk about GWG with women? Do you feel comfortable referring people to the clinic? Why/why not?
Beliefs about consequences (All interviewees)	Can you tell me about your thoughts about healthy weight gain in pregnancy? Is this a priority for you? What is your attitude to the Healthy Pregnancy service? Do you encourage women to go to the Healthy Pregnancy service? How do women receive the information you provide?
Environmental context and resources (All interviewees)	What problems have you encountered within the Healthy Pregnancy service? Could you suggest any improvements to the Healthy Pregnancy service? Do you communicate with other health care providers about GWG? How? There are fewer women being seen by the implementation team lately. Why do you think this is? Do you feel supported and valued as part of the Healthy Pregnancy service team?
Behavioural regulation (All interviewees)	What systems do you think are needed to improve the delivery of appropriate gestational weight gain advice to women?

**Table 2:** Demographic characteristics of interviewees.

Staff Member *	Position	Experience in this Clinic
Midwife	senior senior junior junior	#1 #3 #5: Significant #2 #7: Limited #6: Limited #4: Some
Nurse unit manager (midwife)	senior	#1: Significant #2: Significant
Obstetrician	senior	Significant
Physician	senior	#1: Significant #2: Significant
Health coach	senior	6 months (maternity leave position)
Administration clerk	administration clerk	Significant

* All are female. Experience: significant, >3 years; some, 1–2 years; limited, <1 year. # The # signifies number, the midwives have been identified by a number in the quotes (eg midwife #3) for anonymity.

**Table 3:** Themes and sub-themes mapped to TDF.

Themes and Sub-Themes	Barrier	Enabler	TDF Domain
THEME 1: Health professionals view themselves as part of team to support healthy lifestyle and GWG Sub-themes:			Social/professional role and identity
Staff roles		+	
Team work/communication between staff	+	+	
Staff feedback		+	
THEME 2: Health professionals reported having skills to run a Healthy Pregnancy service Sub-themes:			Skills
Open communication in describing the clinic to women		+	
Develop rapport		+	
Empowering women to be involved		+	
THEME 3: Experience generated confidence in discussing GWG	+	+	Beliefs about confidence
THEME 4: Health professionals want women to be comfortable in this service Sub-themes:			Emotions Behavioural regulation
Women’s reluctance to engage	+		
Midwives’ fear of upsetting women	+		
Managing women’s negative experience		+	
THEME 5: GWG is variably considered of importance among health professionals	+	+	Beliefs about consequences
THEME 6: The environmental context and resources presented some barriers Sub-themes:			Environmental context and resources
Waiting time and clinic day unsuitable for women	+		
Lack of awareness of Healthy Pregnancy service amongst some midwives	+		
Improving patient resources		+	

The + corresponds to the sub-themes (whether they were barriers or enablers).
